# Criticality in the Healthy Brain

**DOI:** 10.3389/fnetp.2021.755685

**Published:** 2022-01-18

**Authors:** Jifan Shi, Kenji Kirihara, Mariko Tada, Mao Fujioka, Kaori Usui, Daisuke Koshiyama, Tsuyoshi Araki, Luonan Chen, Kiyoto Kasai, Kazuyuki Aihara

**Affiliations:** ^1^ International Research Center for Neurointelligence, The University of Tokyo Institutes for Advanced Study, The University of Tokyo, Tokyo, Japan; ^2^ Institute of Industrial Science, The University of Tokyo, Tokyo, Japan; ^3^ Department of Neuropsychiatry, Graduate School of Medicine, The University of Tokyo, Tokyo, Japan; ^4^ Disability Services Office, The University of Tokyo, Tokyo, Japan; ^5^ Center for Excellence in Molecular Cell Science, Shanghai Institute of Biochemistry and Cell Biology, Chinese Academy of Sciences, Shanghai, China; ^6^ Guangdong Institute of Intelligence Science and Technology, Zhuhai, China; ^7^ School of Life Science and Technology, ShanghaiTech University, Shanghai, China; ^8^ Key Laboratory of Systems Biology, Hangzhou Institute for Advanced Study, University of Chinese Academy of Sciences, Chinse Academy of Sciences, Hangzhou, China

**Keywords:** critical brain hypothesis, dynamical network analysis, criticality of the neuronal network, psychotic disorder, schizophrenia, mismatch negativity, risk of mental disorder, dynamical network marker

## Abstract

The excellence of the brain is its robustness under various types of noise and its flexibility under various environments. However, how the brain works is still a mystery. The critical brain hypothesis proposes a possible mechanism and states that criticality plays an important role in the healthy brain. Herein, using an electroencephalography dataset obtained from patients with psychotic disorders (PDs), ultra-high risk (UHR) individuals and healthy controls (HCs), and its dynamical network analysis, we show that the brain of HCs remains around a critical state, whereas that of patients with PD falls into more stable states. Meanwhile, the brain of UHR individuals is similar to that of PD in terms of entropy but is analogous to that of HCs in causality patterns. These results not only provide evidence for the criticality of the normal brain but also highlight the practicability of using an analytic biophysical tool to study the dynamical properties of mental diseases.

## Introduction

Studying the human brain is a large project, which involves investigating its organization, structure, function, and association with behavior. Detecting the physical mechanisms underlying the working of the brain is one of the most important topics. On the one hand, the brain should be well-structured to process information appropriately. On the other hand, it must be flexible enough to adapt to various environments and emergencies.

The critical brain hypothesis provides an intriguing explanation for the mechanism of the working of the brain, which assumes that in the normal brain, neural networks work near a critical state (Beggs, 2015; Massobrio et al., 2015; Beggs and Timme, 2012). Self-organized criticality (Cocchi et al., 2017; Lee et al., 2019) is one argument of the critical brain hypothesis, which has been supported by the neuronal avalanche phenomenon experimentally (Beggs and Plenz, 2003; Petermann et al., 2009): the size of the cortical activities exhibits power laws. Studies have also found a possibility that this critical mechanism in the brain could ensure maximized capacity and transmission of information (Haldeman and Beggs, 2005; Shew et al., 2009; Shew et al., 2011). However, although neuronal avalanches and power laws provide important statistical descriptions of the critical brain, an intrinsic dynamical interpretation is still missing.

The dynamical network analysis or dynamical network marker (DNM) theory has been studied in the recent decade; it provides a dynamics-based tool to detect the changes in a complex system under perturbation, especially near the critical point (Chen et al., 2012; Liu et al., 2012; Kuehn, 2011; Shi et al., 2016). The DNM theory generalizes the approach of detecting early warning signals of critical slowing down phenomena (Scheffer et al., 2001; Scheffer, 2009; Scheffer et al., 2009) to complex networks. According to the DNM theory, a core subnetwork called a dynamical network (DNMnet) can be found, which is the leading subnetwork of the system toward criticality. Its components (called the DNM group) exhibit large deviations in signals and strong correlations between them around the critical state. DNM groups can not only act as a marker for the criticality of complex systems but also provide an approach for predicting disease, economic crashes, etc (Liu et al., 2014a; Liu et al., 2015; Richard et al., 2016; Lesterhuis et al., 2017; Li et al., 2013; Liu et al., 2014b; Yang et al., 2018; Liu et al., 2019; Dakos and Bascompte, 2014).

Herein, we used electroencephalography (EEG) (Cooper et al., 2014) data recorded from healthy control subjects (HCs), ultra-high risk (UHR) individuals, and patients with psychotic disorder (PD) to explore states of the brain using the DNM theory. By analyzing the dynamical properties of the DNM group of the neural network in the brain, we found that brains of HCs were at a critical state, whereas those of patients with PD were stuck in more stable less-critical states. Brains of UHR individuals fell in a medial state with similar low entropy as those of patients with PD, but a weak causality pattern was observed between electrodes similar to HC. These results based on DNM modeling not only provide an evidence of the critical brain hypothesis but also show a biophysical framework to study the brain and mental diseases.

## Results

### Construction of the DNMnet

The 64-channel EEG data were recorded from subjects with the two-tone auditory oddball paradigm (see Methods). Mathematically, we suppose that the dynamics of brain signals under the standard stimulus evolves as follows:

x(t+1)= f(x(t), λ), (1)

where $x={\left(x_{1},x_{2},\ldots ,x_{n}\right)}^{T}\in R^{n}$ is a vector containing signals from all n electrodes, f is assumed to satisfy the existence and uniqueness condition, and *λ* is a bifurcation parameter. Because we assume the invariance of the brain dynamics, the signals y for the deviant stimulus (the deviant tone in two-tone auditory oddball paradigm, see Methods), which is a small perturbation from x , follow the same equation y(t+1)=f(y(t),λ) . Thus, the evolution of the difference process z(t) = y(t)−x(t) can be approximated as follows:

z(t+1)≜y(t+1)−x(t+1)

≈A(λ)(y(t)−x(t))+  ξ(t)

**FIGURE 1:**
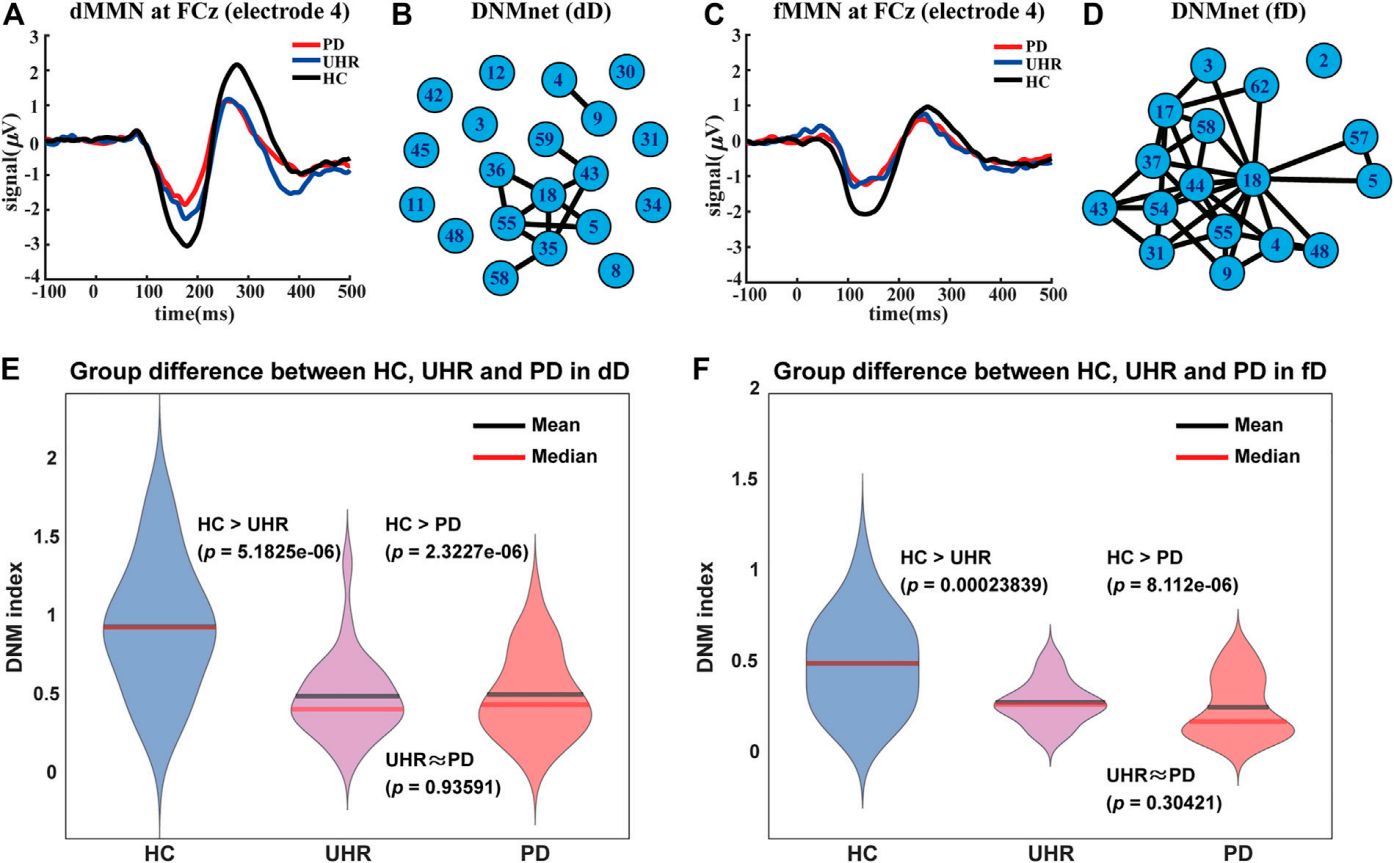
Auditory mismatch negativity (MMN) patterns, dynamical networks (DNMnet), and DNM indices of the three groups (HC, UHR, and PD). **(A)** is the duration MMN (dMMN) pattern and **(C)** is the frequency MMN (fMMN) pattern for the electrode FCz (channel number 4). **(B)** and **(D)** are DNMnets constructed in the duration deviation (dD) case and the frequency deviation (fD) case, respectively. **(B)** has 20 nodes and 12 edges which exhibited significant differences (the one-sided Wilcoxon rank-sum test, $p<10^{-3}$ for nodes and p<0.005 for edges after FDR correction) between the HC and PD groups, and (D) has 17 nodes and 37 edges. For dD in **(E)** and fD in **(F)**, DNM indices were computed, compared, and plotted using violin plots. The p-values were calculated by the one-sided Wilcoxon rank-sum test for “HC > UHR” and “HC > PD”, whereas for “UHR ≈ PD”, the two-sided Wilcoxon rank-sum test was used.

=A(λ)z(t)+ξ(t), (2)

where the matrix $A\left(\lambda \right)\in R^{n\times n}$ is the Jacobian matrix of ***f*** (⋅, λ) , and ξ(⋅) is inevitable noise in the brain and assumed as a small additive noise term which is independent of A(λ) . When the parameter $\lambda \to \lambda_{0}$ , where $\lambda_{0}$ is a codimension-one catastrophic bifurcation point of the system (Kéfi et al., 2013), the largest-modulus eigenvalue of A(λ) will influence system stability. According to the DNM theory (Liu et al., 2012), the network between nodes can be partitioned into a DNM group and a non-DNM group. The DNM group leads the criticality of the system, and deviations of signals and correlations between any nodes in the DNM group will increase sharply around a critical point. Utilizing these characteristics, we constructed the DNMnet and compared the dynamical differences between brains of HCs and those of patients with PD. The DNMnets under duration deviant (dD) experiments and frequency deviant (fD) experiments are shown in Figures 1A–D. Details can be found in the Methods.

### Criticality of the Brain: MMN Pattern and DNMIndex

Mismatch negativity (MMN) (Erickson et al., 2016) is one of the most well-known patterns in PD, whose amplitude of neural activities is reduced compared to the healthy brain. MMN is associated with cognitive impairments (Chung et al., 2017) and can serve as a biomarker for early interventions (Bodatsch et al., 2015) and development of novel treatments (Perez et al., 2017; Kantrowitz et al., 2018). However, mechanisms underlying altered connectivity remain unknown in the clinical field. Using the DNM theory, we found that the MMN amplitude is explained by the difference process z(t) of the brain signal. Furthermore, the nodes in the DNMnet with a large deviation in the signal are all located in the fronto-central area, which corresponds to the region with significant MMN patterns (Figures 1A–D). The DNM theory can provide a dynamical explanation of the MMN pattern, and in return, the MMN pattern provides an evidence for the criticality of the normal brain under the DNM framework.

A DNMIndex was designed to measure the criticality based on the characteristics extracted from the DNMnet as follows:

DNMIndex=Π(σ(x))⋅Π(|ρ|), (3)

where “Π (⋅) ” stands for the geometric mean of a vector, “ σ(⋅) ” is the component-wise standard deviation, “ x ” denotes the brain signals after preprocessing (corrected epochs) of nodes in the DNMnet, “ |⋅| ” is the component-wise absolute value function, and “ ρ ” denotes the correlations between nodes with edges in the DNMnet. The DNMIndex will be higher at the critical state than at non-critical states. Using the DNMIndex, we found the brain of HC is in a significantly more critical state than those of patients with PD and UHR individuals in both dD and fD cases (Figures 1E–F).

### Criticality of the Brain: Entropy

**FIGURE 2:**
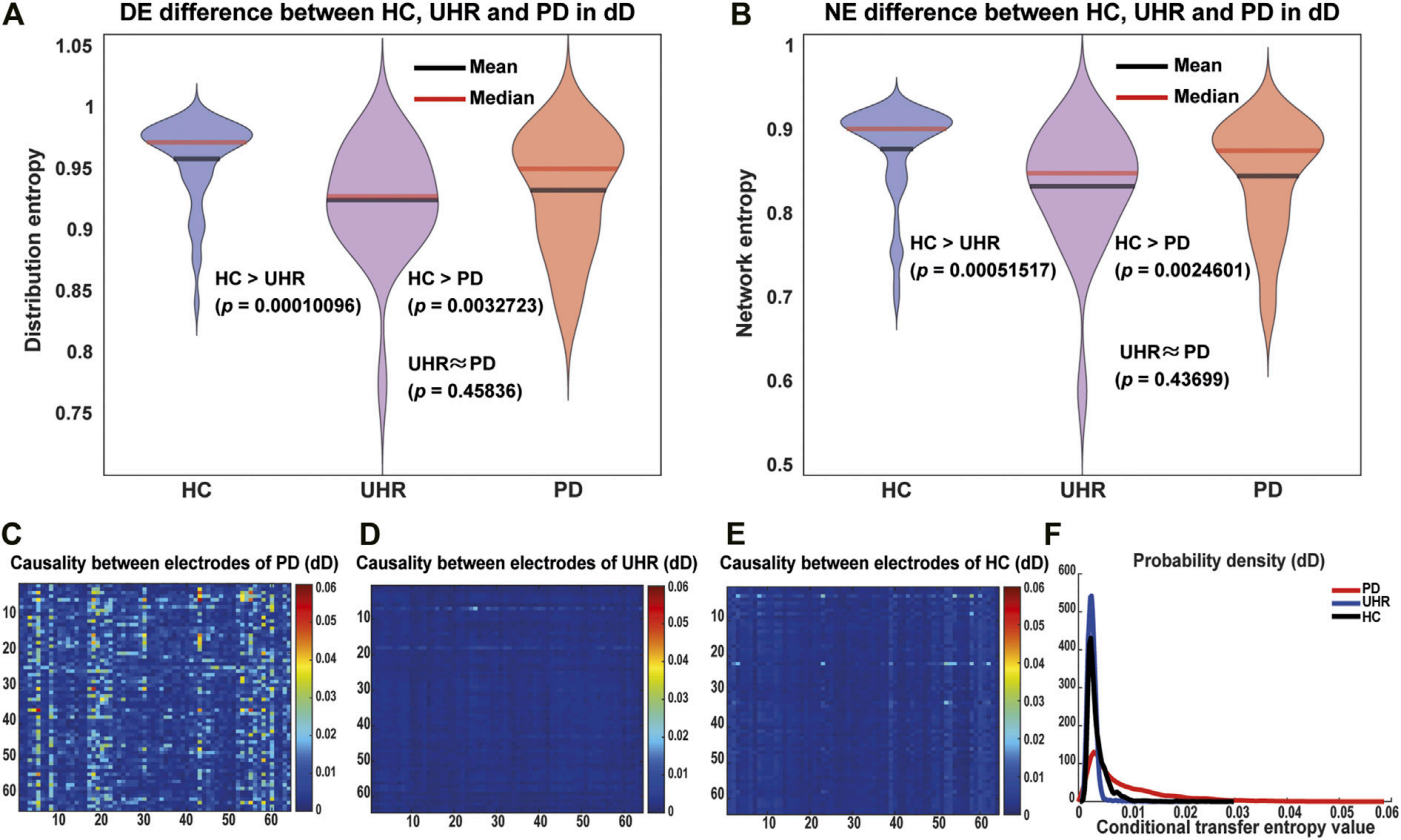
Entropy and causality difference between the HC, UHR, and PD groups in the duration deviant (dD) case. **(A)** Violin plot for the distribution entropy of mutual information between each pair of nodes. **(B)** Network entropy. For “HC > UHR” and “HC > PD”, p-values were calculated by the one-sided Wilcoxon rank-sum test, while for “UHR ≈ PD”, the two-sided Wilcoxon rank-sum test was used. The HC group exhibited significantly higher entropy than the PD and UHR groups. The PD and UHR groups did not significant differences. **(C–E)** Heatmaps of the direct causality between 64 electrodes measured by the conditional transfer entropy (CTE). **(F)** shows probability density functions of the CTE. On average the PD has much stronger direct causalities between electrodes than the HC/UHR.

Mutual information between each pairs of nodes over time and its entropy are important metrics for isotropy and the criticality of a network. Based on the nodes in the DNMnet, we define the mutual information over time from node x to node y as follows:

$$I_{x\to y}=\iint p\left(x_{t},\;y_{t+\Delta t}\right)\log\frac{p\left(x_{t},y_{t+\Delta t}\right)}{p\left(x_{t}\right)p\left(y_{t+\Delta t}\right)}\text{d}x\text{d}y$$ (4)

where $\text{\{}x_{t}\text{|}t\;=\;1,\;2,\;\ldots \}$ and $\text{\{}y_{t}\text{|}t=\;1,\;2,\;\ldots \}$ are the observed time series from node x and y ; Δt is a time interval; $p\left(x_{t},\;y_{t+\Delta t}\right)$ is the joint probability density function; $p\left(x_{t}\right)$ and $p\left(y_{t+\Delta t}\right)$ are the respective marginal density functions; and the integral is over the entire $\left(x_{t},\;y_{t+\Delta t}\right)$ space. According to the DNM theory, if x and y belong to the DNM group, their correlation will become strong near the critical point; thus, $I_{x\to y}$ grows rapidly. However, if either x or y belongs to the non-DNM group, mutual information will remain stable and bounded. Thus, near the critical point, some rapidly growing mutual information will change their distribution over the entire DNMnet. We will use two indices to detect the anisotropy of mutual information in the network—the distribution entropy (DE) and network entropy (NE)—whose definitions are shown in the Methods. With the time interval Δt=20 ms, we calculated the DE and NE of the HC, UHR, and PD groups (Figures 2A–B for the dD case, and Supplementary Figures S7A–B for the fD case). The HC group always exhibited significantly higher DE and NE than the PD and UHR groups, while the difference between the PD and UHR groups was not significant. These results support the critical brain hypothesis that the HC group should be at a critical state, whose entropy is high possibly to ensure adaptivity. In addition, correlations between positive symptoms (clinical score) of PD and DE/NE were calculated (Supplementary Figure S11), which also implied that the worse the positive symptoms are, the more low-entropy and stable the brain is. In Supplementary Figure S12, we also showed that the orders of DE/NE for three groups were not sensitively influenced by the parameter Δt .

In the biological sense, the high entropy at the critical state reflects a possibility that the brain can send information to different brain regions in time to deal with various stimuli.

### Criticality of the Brain: Causality Pattern

The causality pattern is another evidence for criticality, which can be detected by the conditional transfer entropy (CTE) between different nodes, which is given as

$$\mathrm{CT}E_{x\to y}=\mathrm{CMI}\left(x_{t},\;y_{t+\Delta t}\text{|}{\bar{x}}_{t}\right)=\int \int \int p\left(x_{t},\;y_{t+\Delta t},{\bar{x}}_{t}\right)\log\frac{p\left(x_{t},y_{t+\Delta t}|{\bar{x}}_{t}\right)}{p\left(x_{t}|{\bar{x}}_{t}\right)p\left(y_{t+\Delta t}|{\bar{x}}_{t}\right)}\text{d}x\text{d}y\text{d}{\bar{x}}_{t},$$ (5)

where CMI denotes the condition mutual information, *x* and *y* are two variables to detect causality, $\bar{x}$ includes all other variables except *x*, and Δt is the time interval. CTE is different from mutual information because it excludes indirect influences and is usually used to detect the direct causality. If the brain is at a stable state, causalities between different electrodes should be apparently present, while for critical states, random-like activities decrease the possibility of the appearance of regular causality patterns. With the time interval Δt=20 ms, we can obtain heatmaps of the mean CTE between every pair of 64 electrodes for the PD, UHR, and HC groups (Figures 2C–E for the dD case, and Supplementary Figures S7C–E for the fD case). The larger the value of CTE is, the stronger is the direct causality between variables. We found that the PD had a stronger causality network compared with the UHR/HC, which implies that psychosis makes the brain fall into a more stable state. In contrast, it is difficult for the normal brain to form general causality patterns at a critical state, as shown by the heatmap of the HC group. The probability density functions of CTE for each group are plotted in Figure 2F for a better intuition. In Supplementary Figure S12, we also showed that the orders of CTE for three groups were not sensitively influenced by the parameter Δt .

### ANCOVA for DNMIndex, DE, and NE

For EEG data, we performed analysis of covariance (ANCOVA) with age, premorbid IQ, and antipsychotic dose as covariates because these variables were significantly different among groups (Supplementary Table S1). For the duration deviant experiments, all these covariates showed no significant effects (F_1,91_ < 2.58, *p* > 0.11). Therefore, these factors did not affect our findings in the duration deviant experiments. For the frequency deviant experiments, premorbid IQ showed significant effects, whereas age and antipsychotic dose showed no significant effects (F_1,91_ < 0.75, *p* > 0.39). Therefore, we performed ANCOVA with premorbid IQ as a covariate in the frequency deviant experiments.

ANCOVA of the DNMIndex in the frequency deviant condition revealed significant effects of groups (F_2,95_ = 6.61, *p* = 0.01) and premorbid IQ (F_1,95_ = 11.08, *p* < 0.001). Post-hoc analyses revealed that the HC group had a significantly higher mean value than the PD (*p* < 0.001) and the UHR (*p* = 0.004) groups, while the difference between the PD and UHR groups was not significant (*p* = 1.00).

ANCOVA of DE in the frequency deviant condition revealed significant effects of groups (F_2,95_ = 5.72, *p* = 0.005) and premorbid IQ (F_1,95_ = 6.80, *p* = 0.01). Post-hoc analyses revealed that the HC group had a significantly higher mean value than the PD group (*p* = 0.003), while the difference between the HC and UHR groups (*p* = 0.33) and the difference between the UHR and PD groups (*p* = 0.48) were not significant. ANCOVA of NE in the frequency deviant condition revealed significant effects of groups (F_2,95_ = 5.44, *p* = 0.006) and premorbid IQ (F_1,95_ = 8.43, *p* = 0.005). Post-hoc analyses revealed that the HC group had a significantly higher mean value than the PD group (*p* = 0.004), while the difference between the HC and UHR groups (*p* = 0.58) and the difference between the UHR and PD groups (*p* = 0.31) were not significant.

These findings revealed the lower DNMIndex, lower distribution entropy, and lower network entropy of the frequency deviant experiments in the PD group even after controlling premorbid IQ. In UHR, the DNMIndex was lower, whereas DE and NE showed insignificant difference for the frequency deviant experiments after controlling premorbid IQ.

### Application of Criticality: Risk of Mental Disease

**FIGURE 3:**
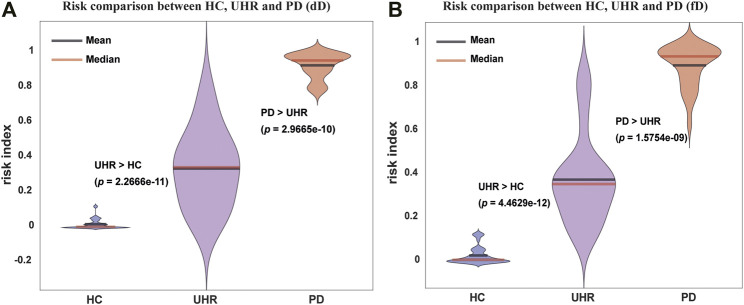
Risk analysis of the HC, UHR, and PD groups for the duration deviant experiment (dD) dataset and the frequency deviant experiment (fD) dataset with DNM features. **(A)** and **(B)** Violin plots of risks for three groups in the dD and fD cases, respectively. The HC always has the lowest and the PD has the highest risk, whereas the UHR exhibits a medial risk. The one-sided Wilcoxon rank-sum test was used to determine the significance of difference between groups.

The level of the criticality of the brain contained in the DNMnet can provide a quantitative measure to weigh the risk of mental disease. From the EEG samples, we applied an ensemble classifier based on the DNMnet (see Methods). AUCs and cross validations were used to test the training accuracy and to ensure the reliability of the classifier (Supplementary Figure S8). With the final risk output, we found that the PD group exhibited the highest risk, and the HC group showed the lowest risk, whereas the UHR group evinced a medial risk (Figure 3A for the dD case and Figure 3B for the fD case). This risk analysis indicates that DNM results could help distinguish between the three groups and has a potential to guide prepsychotic diagnosis.

## Discussion

In this study, we built a dynamical network model to explore the criticality of the brain. It not only provides a dynamics-based explanation of the traditional MMN patterns, but also uses features of the model (the DNMIndex, the entropy, and the causality pattern) to support the fact that the healthy brain is around the critical state. A risk index for PDs also indicates the practicality of the DNMnet, which can highlight the criticality of the neuronal network.

Criticality is an important concept for the activity of the brain. Around the critical state, the healthy brain can adapt to new situations and deal with various stimuli in a timely manner. However, if the DNMnet in the brain falls into a more stable state, as in the PD group, the stability may lead to cognitive inflexibility and functional impairments (Serrano-Guerrero et al., 2020). Previous studies reported altered connectivity underlying MMN in psychotic disorders (Dima et al., 2012; Ranlund et al., 2016; Braeutigam et al., 2018). However, MMN does not highlight the dynamical mechanism to clarify its reduction in psychotic disorders. Therefore, the DNMnet used in the current study reveals that MMN is only an external presentation of the changes in the criticality.

The UHR group in this study showed lower DNMIndex, DE, and NE compared to the HC group. These findings suggest that the DNMnet of the UHR has already fallen into the stable states before onset of psychosis. However, in the direct causality network constructed by the CTE, we found that the direct causality between electrodes for the UHR group was as weak as the HC. As the CTE detects the direct influence from electrode to electrode, while the DE and NE consider the long-range global communication, we speculate a possibility that the UHR group has healthy local functions similar to the HC group, but impaired global functions similar to the PD group. The lack of variation may eventually pull the UHR into a more stable state, similar to the PD group. It could be one way to explain why the brain of UHR individuals works normally as HC compared to those of PD, but eventually the slowing down variation makes it fall into the stable PD state.

In the application of risk analyses, we only used the information from the DNMnet, which is a subnetwork of the entire 64 channels, to estimate the risk of psychosis for each sample. We also compared it with the result of the risk estimated by the MMN amplitude (Supplementary Table S4; Supplementary Figures S9, S10). The DNMnet exhibited the same efficiency as the MMN risk which uses features from all channels. These findings indicate that the much simpler DNMnet can serve as not only a mechanistic biomarker (Pine and Leibenluft, 2015) but also as a pragmatic biomarker (Paulus, 2015) for diagnosing mental diseases.

There are several limitations in this study. First, potential medication effects may have influenced the findings because participants in UHR or PD took medication. Whether medication biases dynamic network markers needs to be clarified with medication-naïve participants in the future study. Second, this work is designed as a cross-sectional study. Therefore, we could not identify when the brain falls into stable states. Future longitudinal studies will clarify the trajectory of dynamic states in psychotic disorders.

In conclusion, we found that the criticality is a key feature of the healthy brain. Based on the DNM theory, the brain of HC was close to the critical states, whereas those of UHR individuals or patients with PD fell into more stable states. Our analysis not only offers a new viewpoint toward understanding the dynamic brain but also provides a possibility of a biophysical approach for researching other mental disorders.

## Methods

### Subjects

A total of 49 HC subjects, 24 UHR individuals, and 29 patients with PD participated in this study, which contained participants of the Integrative Neuroimaging Studies for Schizophrenia Targeting Early Intervention and Prevention (IN-STEP) (Koike et al., 2013). Detailed participant information is provided in the Supplementary Notes and Supplementary Table S1. The Research Ethics Committee of the Faculty of Medicine, The University of Tokyo, approved this study (approval No. 629, 2226). We conducted this study in accordance with the Declaration of Helsinki. Written informed consent was obtained from all the participants.

### Electroencephalography Data

A 64-channel Geodesic EEG System (Electrical Geodesics Inc, Eugene, OR) was used to acquire EEG data. Electrodes were referenced to the vertex, and impedances were kept below 50 kΩ. The sampling rate was 500 Hz, and the analog filter bandpass was set at 0.1–100 Hz. The locations of the EEG electrodes are shown in Supplementary Figure S1A.

### Stimuli and Procedure

The two-tone auditory oddball paradigm with 2000 stimuli was performed for each subject when obtaining EEG data. For the duration deviant (dD) experiments, 90% stimuli were standard tones (1,000 Hz, 50 ms) and 10% were deviant tones (1,000 Hz, 100 ms). For the frequency deviant (fD) experiments, 90% stimuli were standard tones (1,000 Hz, 50 ms) and 10% were deviant tones (1,200 Hz, 50 ms). All stimuli were 80 dB SPL with a 1 ms rise/fall time. The stimulus onset asynchrony was 500 ms. The oddball paradigms were counter-balanced, and tones were presented binaurally through earphones while participants watched a silent cartoon.

### EEG Data Preprocessing

Original 64-channel signal files (Supplementary Figure S1B) were input into MATLAB (9.3.0) and were further preprocessed using the EEGLAB (v14_1_1b) package (Delorme and Makeig, 2004) (Supplementary Figures S1C–E). Detailed preprocessing procedures are provided in the Supplementary Material. Finally, corrected epochs (deviant epochs with the mean signal of standard epochs being subtracted for each sample) were utilized for further analyses (Supplementary Figure S1F).

### Auditory Mismatch Negativity

Corrected epochs were obtained by subtracting the event-related potential (ERP) waveforms in response to the standard stimuli from those in response to the deviant stimuli. We defined the peak latency as the most negative peak between 100 and 250 ms relative to the onset, and the MMN amplitude was calculated as the mean signal around the peak latency (we used the window of 135–205 ms for the duration MMN and the window of 100–200 ms for the frequency MMN) (Nagai et al., 2013). The MMN patterns for the 64 electrodes in the three groups (PD, UHR, and HC) under dD and fD are shown in Supplementary Figures S3 and S4, respectively. Using the MMN amplitude for each channel as the feature, we can compute p-values of the group difference under the Wilcoxon rank-sum test. After FDR correction, we identified 12 electrodes (Nos. 3, 4, 5, 9, 17, 18, 22, 30, 43, 54, 55, and 58) in the dD and 12 electrodes (Nos. 3, 4, 5, 8, 9, 17, 18, 30, 43, 54, 55, and 58) in the fD that exhibited significant difference (adjusted *p* < 0.05) between the HC and UHR/PD groups, albeit insignificant difference (adjusted *p* > 0.05) between the UHR and the PD groups. It should be noticed that all electrodes with significant MMN difference were located in the fronto-central area.

### Dynamical Network (DNMnet)

The neuronal signal under standard stimuli is supposed to obey the dynamics in Eq. 1, whereas the corrected epochs after preprocessing follow Eq. 2. Criticality is defined as when the dynamics is around a codimension-1 local bifurcation (Chen et al., 2012). The network of electrodes can be partitioned into a DNM group and a non-DNM group (Chen et al., 2012) by measuring the fluctuations of signals and their correlations between electrodes for each sample. The DNMnet is the leading network for complex systems that drives the system toward or away from critical states. Two conditions for constructing the DNMnet were used in this study:

1. Any member of the nodes in DNMnet is highly fluctuating around a critical point.
2. Any edge in the DNMnet becomes very strong around the critical state.

The one-sided paired-sample *t*-test and the one-sided Wilcoxon rank-sum test were used for selected nodes and edges, respectively. We selected nodes and edges with significant statistical difference between HC and PD groups. Significance was set at $p<10^{-3}$ for nodes and p<0.005 for edges after FDR correction. Supplementary Tables S2, S3 and Supplementary Figures S5, S6 show the statistical properties of the nodes and edges in DNMnets. Specifically, 20 nodes (the one-sided paired-sample *t*-test with $p<10^{-3}$ after FDR correction) were extracted from the dD experiment dataset (Supplementary Figure S5). 12 edges with significantly increasing correlations (the one-sided Wilcoxon rank-sum test with  p<0.005 after FDR correction) were also detected (Supplementary Figure S6). No edge with significantly decreasing correlation (the one-sided Wilcoxon rank-sum test with significance level α=0.05 ) in HC was found. We denoted the 20 nodes and 12 edges as the DNMnet for the dD case (Figure 1B). The same analysis was also applied to the fD experiment dataset, from which 17 nodes (the one-sided paired-sample *t*-test with $p<10^{-3}$ after FDR correction, Supplementary Table S2) and 37 edges (the one-sided Wilcoxon rank-sum test with p<0.005 after FDR correction, Supplementary Table S3) were selected to form the DNMnet for the fD case (Figure 1D). No significant decreasing correlation (the one-sided Wilcoxon rank-sum test with significance level α=0.05 ) in HC was found in the fD experiment dataset, either.

We remark that the Lyapunov Exponent is also a possible measure for detecting criticality. We note that the dynamical network we used here refers to the leading subnetwork (Chen et al., 2012). In the celebrated paper on synchronization (Arenas et al., 2008), the dynamical network is considered as the whole system of the interacting dynamical units. In this manuscript, most of the time we use the notation the DNMnet to avoid the confusion.

### Measures for the Criticality

When calculating the DNMIndex using Eq. 3, the corrected epochs were used as the signal. Nodes and edges in the DNMnet were considered.

We denote a network as G = {V, E} , where $V\;=\;\left\{x_{1},\;x_{2},\;\ldots ,\;x_{n}\right\}$ are n nodes and $E\;=\;\text{\{}e_{i,j}\text{|}i,\;j\in \;\left\{1,\;2,\;\ldots ,\;n\right\}\;\text{and}\;i\neq \;j\}$ are $N_{e}\;=\;n\left(n-1\right)$ different directed edges. Using the mutual information in Eq. 4, the distribution entropy (DE) is defined as the normalized entropy of all mutual information on edges, as follows:

$${\text{Ent}}_{\text{d}}\;=\;-\frac{1}{\log N_{e}}\overset{n}{\underset{i=1}{\sum }}\overset{n}{\underset{\begin{array}{c} j=1\\ j\neq i \end{array}}{\sum }}p_{ij}\log p_{ij},$$ (6)

where

$$p_{ij}=\frac{I_{x_{i}\to x_{j}}}{\sum_{i=1}^{n}\sum_{j=1,\;j\neq i}^{n}I_{x_{i}\to x_{j}}}$$ (7)

is the normalized mutual information over the entire network. Mutual information $I_{x_{i}\to x_{j}}$ is calculated under Gaussian approximation as ${\text{I}}_{\text{x}\to \text{y}}=\;-1/2\cdot \text{log}\left(1-\rho_{x_{t}y_{t+\text{Δ}t}}^{2}\right)\;$ , where ${\text{ρ}}_{\text{xy}}$ is the Pearson correlation coefficient between x and y. In contrast, the network entropy (NE) is defined as follows

$${\text{Ent}}_{\text{n}}=\;-\frac{1}{n}\overset{n}{\underset{i=1}{\sum }}\overset{n}{\underset{\begin{array}{c} j=1\\ j\neq i \end{array}}{\sum }}q_{ij}\log q_{ij},$$ (8)

where

$$q_{ij}=\frac{I_{x_{i}\to x_{j}}}{\sum_{j=1,\;j\neq i}^{n}I_{x_{i}\to x_{j}}}$$ (9)

is the normalized mutual information exiting each node. We use the full network between nodes in DNMnet to calculate the DE and NE. If the brain works around a critical state, some increasing mutual information will expand their distribution density on the network. Thus, DE and NE are larger at a critical state than at non-critical states. High entropy also reflects a possibility that the brain can send information to different brain regions in time to deal with various stimuli.

For the CTE defined in Eq. 5, every pair between 64 electrodes is considered to construct the causality patterns in (Figures 2C–E). CTE is also calculated under Gaussian approximation as ${\text{CTE}}_{\text{x}\to y}=\;-1/2\cdot \text{log}\left(1-{\text{ρ}}_{x_{t}y_{t+\text{Δ}t|{\bar{x}}_{t}\;}}^{2}\right)$ , where ${\text{ρ}}_{\text{xy}|\text{z}}$ is the partial correlation between x and y conditional on z.

### Estimation of Psychotic Risk

Using the knowledge of the DNMnet after dynamical network analysis, we applied an ensemble classification on the EEG data to estimate the risk index for psychosis. A flowchart of the risk estimation is shown in Supplementary Figure S2.

We assume that there are $n_{k}$ corrected deviant epochs after preprocessing for sample *k*, which may belong to one of the PD, HC, or UHR groups. PD and HC epochs are used as the training set. Features for training comprise the elements in the covariance matrix for each sample. We assigned each epoch a label: 1 for PD and 0 for HC. Ensemble classification was then applied, and cross validation was performed. Using the classification tree, we can test all PD, HC, and UHR epochs. Finally, the risk for person *k* is defined as the mean output (the label value) of the epochs belonging to this sample, and this is defined as

$$R_{k}\;=\frac{1}{n_{k}}\overset{n_{k}}{\underset{i=1}{\sum }}O_{i},$$ (10)

where $R_{k}$ is the risk for sample *k*, $n_{k}$ is the number of corrected epochs in sample *k*, and $O_{i}$ is the classification output of the *i*th epoch. $R_{k}$ is always between 0 and 1. To verify the accuracy and compare different classifiers, we used a risk accuracy index. If there are $n_{\text{HC}}$ samples in HC, $n_{\text{UHR}}$ samples in UHR, and $n_{\text{PD}}$ samples in PD, we sort the risks for all samples in an ascending order from smallest to largest. The risk accuracy for the HC group is set as the percent of HC samples in the first $n_{\text{HC}}$ smallest $R_{k}$ s. The risk accuracy for the UHR group is set as the percent of UHR samples in the medial $n_{\text{UHR}}$ medium $R_{k}$ s. The risk accuracy for the PD group is set as the percent of PD samples in the last $n_{\text{PD}}$ largest $R_{k}$ s. Furthermore, the risk accuracy for the disease group is set as the percent of UHR and PD samples in the last $n_{\text{UHR}}+n_{\text{PD}}$ as the largest $R_{k}$ . The training accuracy of the classifier is defined as 1−loss . Thus, the DNM classifier has more than 80% training accuracy (Supplementary Table S4). The risk accuracy indices for the test set were also calculated and are listed in Supplementary Table S4.

### Statistical Analyses

We used SPSS (IBM Corp., New York, United States) and MATLAB 9.3.0 (MathWorks Inc.) for statistical analyses. For demographic and clinical data, we performed a chi-squared test, independent t-tests, and analysis of variance (ANOVA) for comparison among groups. Bonferroni correction was performed in post-hoc analyses of ANOVA. For EEG data, we performed paired-sample t-tests and Wilcoxon rank-sum tests to compare difference in the MMN, DNM measures, and risks between different groups. When we quantified an alternative hypothesis of a one-side inequality, such as some index with HC > PD, we used the one-side Wilcoxon rank-sum tests. While we quantified an alternative hypothesis of a two-side inequality, such as some index with UHR≠PD (i.e., UHR < PD or UHR > PD), we used the two-side Wilcoxon rank-sum tests. False discovery rate (FDR) was also controlled for multiple comparisons (Benjamini and Hochberg, 1995). Analysis of covariance (ANCOVA) with age, premorbid IQ, and antipsychotic dose as covariates were also conducted, which showed that they did not affect the difference of measures for criticality between groups.

## Data Availability

The original contributions presented in the study are included in the article/Supplementary Material, further inquiries can be directed to the corresponding authors.
