# Supplementary material for: Criticality in the Healthy Brain
Source: Front Netw Physiol. 2022 Jan 18;1:755685. doi: 10.3389/fnetp.2021.755685 (PMC10013033; doi:10.3389/fnetp.2021.755685)
Supplement: Supplementary file 1 [file DataSheet1.pdf]

Supplementary Material for “Criticality in the Healthy Brain”

Jifan Shi[1,2,*] , Kenji Kirihara[3,4], Mariko Tada[3], Mao Fujioka[3], Kaori Usui[3], Daisuke Koshiyama[3], Tsuyoshi Araki[3], Luonan Chen[5,6,7,8], Kiyoto Kasai[1,3,*] , Kazuyuki Aihara[1,2,*]

1 International Research Center for Neurointelligence, The University of Tokyo Institutes for Advanced Study, The University of Tokyo, 113-0033 Tokyo, Japan

2 Institute of Industrial Science, The University of Tokyo, 153-8505 Tokyo, Japan

3 Department of Neuropsychiatry, Graduate School of Medicine, The University of Tokyo, 113-8655 Tokyo, Japan

4 Disability Services Office, The University of Tokyo, 113-8654 Tokyo, Japan

5 Center for Excellence in Molecular Cell Science, Shanghai Institute of Biochemistry and Cell Biology, Chinese Academy of Sciences, 200031 Shanghai, China

6 Center for Excellence in Animal Evolution and Genetics, Chinese Academy of Sciences, 650233 Kunming, China

7 School of Life Science and Technology, ShanghaiTech University, 201210 Shanghai, China

8 Key Laboratory of Systems Biology, Hangzhou Institute for Advanced Study, University of Chinese Academy of Sciences, Chinse Academy of Sciences, 310024 Hangzhou, China

Email: shijifan@ircn.jp; kasaimd@gmail.com; aihara@sat.t.u-tokyo.ac.jp.

1 Supplementary Notes

1.1 Terminology

- Bifurcation/critical point: For a dynamical system, a small change in parameters at the bifurcation point will cause a sudden topological change (usually the stability of an equilibrium) of the system. The real part of the largest eigenvalue will change its sign for the co-dimension one bifurcation.
- Critical state: In thermodynamics, a critical state is defined as the point of phase transition. In this study, we use it as a small neighborhood around the critical point.
- Critical slowing down: The critical slowing down phenomenon means that after a small perturbation of a system, it takes longer time to return to the equilibrium if the system tends towards a critical point.

- Catastrophic bifurcation: Catastrophic bifurcation means a stable state becomes unstable and shows a discontinuous jump to another stable state when transiting across the bifurcation point by changing system parameters. If a stable state continuously splits into others, such as the pitch-fork bifurcation, it is called a non-catastrophic bifurcation.
- Power law: If $y = ax^{-k}$, we say the variable y obeys the power law of variable x. If a distribution $p(x) = Cx^{-k}$ for sufficiently large x, we call it a power law distribution.
- Neuronal avalanche phenomenon: The neuronal network has a cascade of bursts of activity lasting tens of milliseconds, and then, a quiescence period lasting several seconds follows. The size of bursts can be approximated by a power law.
- MMN pattern: Mismatch negativity (MMN) is an auditory event-related potential that occurs when a sequence of repetitive sounds is interrupted by an occasional “oddball” sound that differs in frequency or duration.
- DNM network (DNMnet): It is a subnetwork selected by the dynamical network marker (DNM) analysis. It is the leading network that drives the system toward or away from the critical state.
- DNM index: An index designed in the DNM analysis to measure the criticality of a system.
- Entropy: Entropy is an expression used to describe the randomness of the system. In this study, we used distribution entropy (DE) and network entropy (NE) to measure the disorder of the brain network and conditional transfer entropy (CTE) to measure the direct information transfer (direct causality) between channels.
- DNM risk: It is a risk index designed by ensemble classification from DNM features in DNM network to measure the degree of psychotic disorder.

1.2 Subjects

A total of 49 healthy control subjects (HC), 24 ultra-high risk (UHR) individuals, and 29 patients with psychotic disorder (PD) participated in this study, which included participants of Integrative Neuroimaging Studies for Schizophrenia Targeting Early Intervention and Prevention (IN-STEP)[1]. Out of these 102 samples, 83 subjects participated in our previous MMN studies[2,3,4,5,6]. HC subjects were recruited through advertisements at several universities in Tokyo. UHR individuals and patients with PD were recruited from outpatient and inpatient units at the University of Tokyo Hospital. Inclusion criteria of HC were no personal history of psychiatric disease or a family history of axis I disorders in first-degree relatives. Inclusion criteria of UHR were diagnosis of UHR using the Structured Interview for Prodromal Symptoms (SIPS)[7], age 15-30 years, and no history of antipsychotic drug treatment for more than 16 cumulative weeks. The inclusion criterion of PD was diagnosis of schizophrenia or other psychotic disorders using the Diagnostic and Statistical Manual of Mental Disorders, Fourth Edition (DSM-IV)[8]. Exclusion criteria of all groups were neurological illness, traumatic brain injury with any known cognitive consequences or loss of consciousness for more than 5 minutes, history of electroconvulsive therapy, low premorbid intelligence quotient (IQ; below 70), previous alcohol/substance abuse or addiction, and hearing impairment assessed with a hearing test in both ears at 30-dB sound pressure level (SPL) tone at 1000 Hz and 40-dB SPL at 4000 Hz by audiometer. In the PD group, 21 patients had schizophrenia, 6 patients had schizophreniform disorder, 1 patient had delusional disorder, and 1 patient had psychotic disorder not otherwise specified. We selected patients with PD rather than patients with schizophrenia because previous studies revealed that reduced MMN amplitude was not specific to schizophrenia but common to psychotic disorders[3,4]. Twelve of 24 UHR individuals and 26 of 27 patients with PD took antipsychotic medication (Medication data of 2 patients with PD were missing). Written informed consent was obtained from each subject before participation.

The estimated premorbid IQ was assessed using the Japanese version of the National Adult Reading Test in all participants[9,10]. The Positive and Negative Syndrome Scale (PANSS)[11] and the Global Assessment of Functioning (GAF)[8] were used to measure clinical symptoms and global functioning, respectively, of all participants with UHR or PD. The daily dose of antipsychotic medication was converted to the chlorpromazine equivalent dose[12]. The Research Ethics Committee of the Faculty of Medicine, The University of Tokyo, approved this study (approval No. 629, 2226).

1.3 Demographics and clinical characteristics

Table S1 shows demographics and clinical characteristics. In the PD group, 21 patients had schizophrenia, 6 patients had schizophreniform disorder, 1 patient had delusional disorder, and 1 patient had psychotic disorder not otherwise specified.

1.4 EEG data preprocessing

Original 64-channel signal files (Figure S1(B)) were input into MATLAB (9.3.0) and further preprocessed (Figure S1(C-E)) using the EEGLAB (v14_1_1b) package[13]. The following procedures were performed successively. 1) The signal data were filtered and only the frequency between 1 and 55 Hz were reserved (FIR bandpass filter). Then, a notch filter was applied to exclude 49-51 Hz because the signal-to-noise ratio of the data around 50 Hz was very low due to the alternating current frequency. 2) The data were cleaned using the Artifact Subspace Reconstruction (ASR) plugin in EEGLAB[14]. We used the default parameter setting while turning off the bandpass filter in ASR. ASR can remove channels with large noise, repair windows of signal with bursts, and remove abnormal components in EEG data, such as eye and muscle movement. 3) Channels that had been removed because of their excessive noise were interpolated. 4) Data were re-referenced to their average signal using the FullRankAverRef plugin. 5) Date were sliced into epochs, with each epoch covering an interval from −100 to 500 ms around the stimulus whose beginning was set at 0 ms. 6) Epochs with any signal value exceeding ±100 μV at any electrode were rejected as likely artifacts. 7) Baseline correction was performed to generate −100 to 0 ms data with zero mean for each epoch. 8) The mean signal of standard epochs was subtracted from each deviant epoch for each sample. Then, the corrected deviant epochs were utilized for further analyses (Figure S1(F)). We list the code for the preprocessing of EEG data in the dD case in the following.

```
%% Preprocessing of EEG data in dD experiment
EEG = pop_readegi(folder, filename), [], [], 'auto');
a = {'dsta', 'ddev'}; %types of stimulus
%EEG is the loaded data
EEG = eeg_checkset( EEG );
%EGI64.ced contains the location of electrodes
EEG=pop_chanedit(EEG, 'load',{'EGI64.ced' 'filetype'
'autodetect'},'changefield',{65 'datachan' 0});

locfile = EEG.chanlocs;
EEG = eeg_checkset( EEG );
%FIR filter
EEG = pop_eegfiltnew(EEG, 1,55,[],0,[],0);
EEG = eeg_checkset( EEG );
EEG = pop_eegfiltnew(EEG, 49,51,[],1,[],0);
EEG = eeg_checkset( EEG );
%Clean data by ASR plugin
EEG = clean_rawdata(EEG, 5, 'off', 0.8, 4, 20, 0.5, 'availableRAM_GB', 7.5);
%Interpolation of the removed channels
```

```
EEG = pop_interp(EEG, locfile, 'spherical');
%Re-reference to the average signal by FullRankAveRef plugin
EEG = fullRankAveRef(EEG);
EEG = eeg_checkset( EEG );

%Slice signal with standard stimulus into epochs and remove artifacts
EEG2 = pop_epoch( EEG, a(1), [-0.1, 0.5], 'newname', 'EGI sta', 'valuelim', [-100
100], 'epochinfo', 'yes');
EEG2 = eeg_checkset( EEG2 );
%Baseline correction
EEG2 = pop_rmbase( EEG2, [-100    0]);
EEG2 = eeg_checkset( EEG2 );
out1 = EEG2.data;

%Slice signal with deviant stimulus into epochs and remove artifacts
EEG3 = pop_epoch( EEG, a(2), [-0.1, 0.5], 'newname', 'EGI dev', 'valuelim', [-100
100], 'epochinfo', 'yes');
EEG3 = eeg_checkset( EEG3 );
%Baseline correction
EEG3 = pop_rmbase( EEG3, [-100    0]);
EEG3 = eeg_checkset( EEG3 );
out2 = EEG3.data;

ddev = out2; dsta = out1;
%save ddev and dsta
```

2 Supplementary Tables and Figures

Table S1: Demographics and clinical characteristics of participants

	HC	UHR	PD	Statistics
Sex (Male/Female)	27/22	16/8	22/7	$\chi^2 = 3.51$, df = 2, p = 0.17
Age (years)[a]	25.7 (6.3)	20.1 (3.8)	25.6 (6.5)	$F_{2,99} = 8.15$, p = 0.001*
Premorbid IQ[b]	109.7 (8.2)	104.2 (7.3)	106.1 (9.4)	$F_{2,96} = 3.85$, p = 0.02*
DOI (months)			34.9 (59.5)	
PANSS				
Positive		14.4 (3.3)	15.0 (4.5)	$t_{51} = -0.47$, p = 0.64
Negative		19.0 (5.5)	18.4 (6.4)	$t_{51} = 0.33$, p = 0.74
General		37.5 (7.3)	33.7 (8.1)	$t_{51} = 1.78$, p = 0.08
Total		70.9 (13.9)	67.1 (17.1)	$t_{51} = 0.88$, p = 0.39
GAF		49.2 (10.8)	43.6 (13.5)	$t_{51} = 1.64$, p = 0.11
Antipsychotic dose (mg/day)[c]		213 (355)	436 (291)	$t_{49} = -2.46$, p = 0.02

*All values except for sex are shown as mean (standard deviation). * indicates significance (p < 0.05). Abbreviations: HC, healthy control; UHR, ultra-high risk; PD, psychotic disorders; IQ, intelligence quotient; DOI, duration of illness; PANSS, positive and negative syndrome scale; GAF, global assessment of functioning.*

[a] *HC > UHR (p = 0.001), UHR < PD (p = 0.003).*

[b] *Missing data of 3 HC. HC > UHR (p = 0.03).*

[c] *Missing data of 2 PD.*

Table S2: p-values of the 17 electrodes with significantly increasing standard deviations in the HC group compared to those in the PD group. The experiment was conducted under frequency deviant (fD) stimuli. The p-values were calculated using one-sided paired-sample T-test with FDR correction. Nodes were selected at $p < 10^{-3}$.

Chan No.	*p*-value	Chan No.	*p*-value	Chan No.	*p*-value
2	5.4958E-04	18	2.8227E-04	54	2.5715E-04
3	5.1810E-04	31	5.7009E-04	55	2.7972E-04
4	2.3473E-04	37	7.2300E-04	57	2.2328E-04
5	7.3318E-05	43	2.2862E-04	58	2.3572E-04
9	3.1143E-04	44	7.0899E-04	62	7.4884E-04
17	2.5275E-04	48	3.9554E-04		

Table S3: p-values of 37 edges with significantly increasing correlations in the HC group compared to those in the PD group. The experiment was conducted under frequency deviant (fD) stimuli. The p-values were calculated using the one-sided Wilcoxon rank-sum test with FDR correction. Edges were selected at $p < 0.005$.

(ChanX, ChanY)	*p*-value	(ChanX, ChanY)	*p*-value	(ChanX, ChanY)	*p*-value
(17, 3)	1.5473E-03	(54, 17)	2.5126E-03	(54, 31)	2.1365E-03
(18, 3)	3.7381E-04	(58, 17)	3.8968E-03	(55, 31)	2.8659E-03
(9, 4)	4.8967E-03	(62, 17)	2.0910E-03	(43, 37)	2.6590E-03
(18, 4)	2.2626E-03	(31, 18)	2.5907E-03	(55, 37)	3.8968E-03
(44, 4)	3.3456E-03	(37, 18)	3.7381E-04	(58, 37)	2.2302E-03
(48, 4)	4.3835E-03	(44, 18)	3.7381E-04	(44, 43)	2.2626E-03
(18, 5)	9.9846E-04	(48, 18)	1.4752E-03	(54, 43)	2.6590E-03
(57, 5)	3.3212E-03	(54, 18)	1.0608E-03	(54, 44)	2.6590E-03
(18, 9)	2.1799E-03	(55, 18)	2.1349E-03	(55, 44)	2.1365E-03
(54, 9)	3.3318E-03	(57, 18)	9.9846E-04	(58, 44)	2.1349E-03
(55, 9)	4.3547E-03	(58, 18)	2.1349E-03	(55, 48)	2.6590E-03
(37, 17)	2.6590E-03	(62, 18)	3.7381E-04		
(44, 17)	2.1349E-03	(43, 31)	2.1365E-03		

Table S4: Summary of measures for the risk classifier

		dD		fD	
		DNM classification	MMN classification	DNM classification	MMN classification
Training set	AUC	0.9875	0.9958	0.9965	0.9981
	Holdout5% loss	0.1585	0.3409	0.1656	0.3417
	Holdout5% accuracy	0.8415	0.6591	0.8344	0.6583
	20fold loss	0.1572	0.3316	0.1789	0.3368
	20fold accuracy	0.8428	0.6684	0.8211	0.6632
Test set	*p*-value (HC < UHR)	2.267e-11	6.443e-12	4.463e-12	3.946e-12
	p-value (UHR < PD)	2.967e-10	2.659e-10	1.575e-09	5.230e-10
	accHC	0.9388	0.9592	0.9592	0.9592
	accUHR	0.8333	0.9167	0.8333	0.8333
	accPD	0.9655	1.0000	0.9310	0.9310
	accDis	0.9434	0.9623	0.9623	0.9623

**dD, duration deviant experiment dataset; fD, frequency deviant experiment dataset. The p-values were calculated using the one-sided Wilcoxon rank-sum test. accHC, risk accuracy for the HC group; accUHR, risk accuracy for the UHR group; accPD, risk accuracy for the PD group; accDis, risk accuracy for the disease group (UHR+PD). The DNM classifier exhibited better training accuracy than the MMN classifier, while both performed well on the test set.*

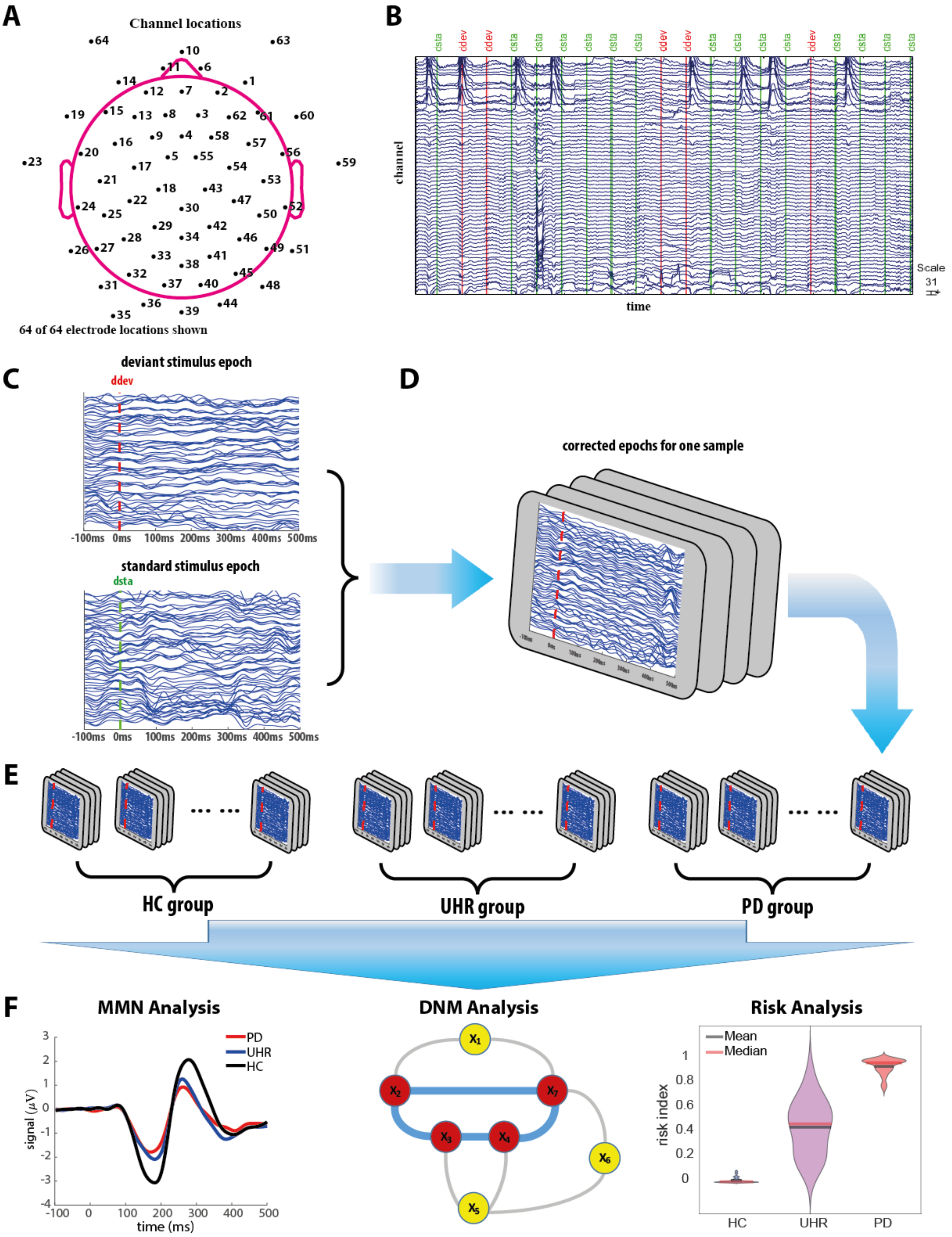
A
Channel locations
64 of 64 electrode locations shown
B
channel
time
Scale
31
C
deviant stimulus epoch
ddev
-100ms 0ms 100ms 200ms 300ms 400ms 500ms
standard stimulus epoch
dsta
-100ms 0ms 100ms 200ms 300ms 400ms 500ms
D
corrected epochs for one sample
E
HC group
UHR group
PD group
F
MMN Analysis
PD
UHR
HC
signal (μV)
time (ms)
DNM Analysis
Risk Analysis
Mean
Median
risk index
HC
UHR
PD

Figure S1: Overview of the EEG data and analysis procedures. (A) 64-channel locations of the EEG measurement. (B) 64-channel EEG signals measured under the two-tone auditory oddball paradigm. (C-F) Flowchart of our EEG dataset analysis procedure. (C) Epochs that were extracted from the original dataset. (D) The sample for one individual that contained numerous corrected epochs. (E) Three groups were evaluated: healthy controls (HC), ultra-high risk (UHR) individuals, and patients with psychotic disorder (PD). (F) Mismatch negativity (MMN) analysis, dynamical network marker (DNM) analysis, and risk analysis were applied to the data.

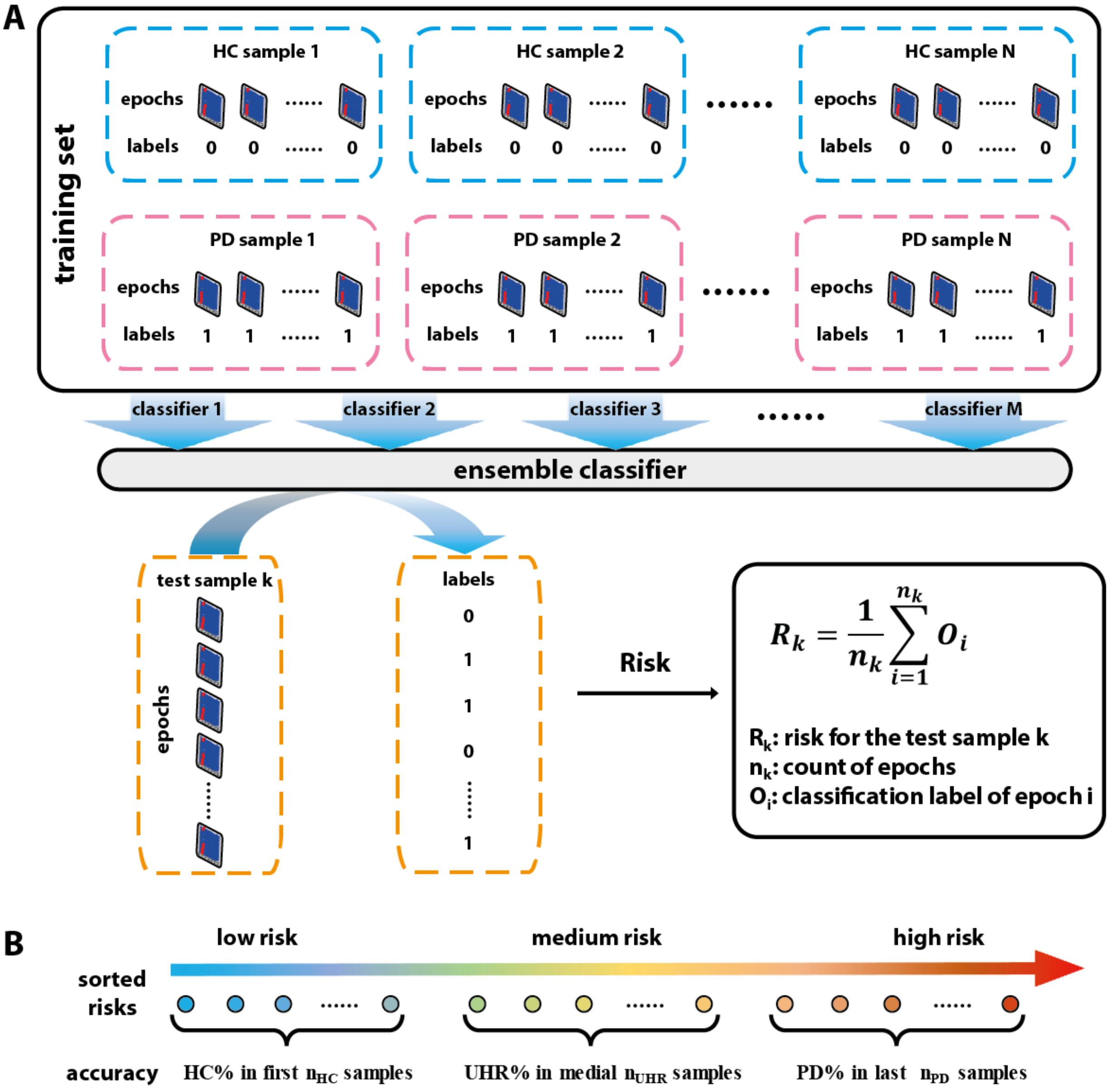

Figure S2: Procedures for risk analysis. (A) Workflow of the risk estimation by the ensemble classification. The risk for each sample is set as the mean value of the output labels for the epochs. (B) Illustration of the risk accuracy defined for the healthy controls (HC), ultra-high risk individuals (UHR), and patients with psychotic disorder (PD).

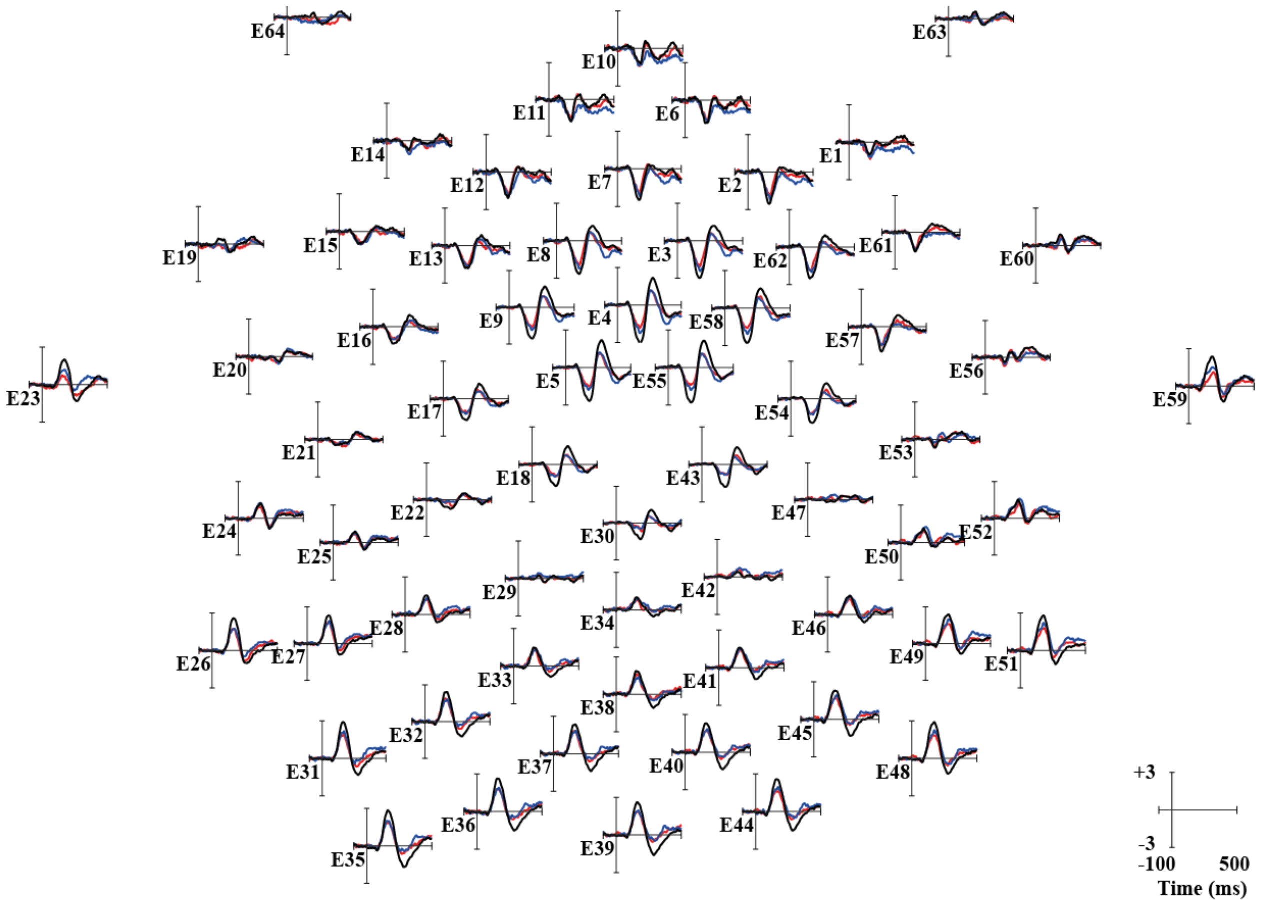

Figure S3: Duration mismatch negativity (dMMN) at all 64 channels of the three groups. Black, blue, and red curves indicate healthy controls (HC), ultra-high risk (UHR) individuals, and patients with psychotic disorders (PD), respectively.

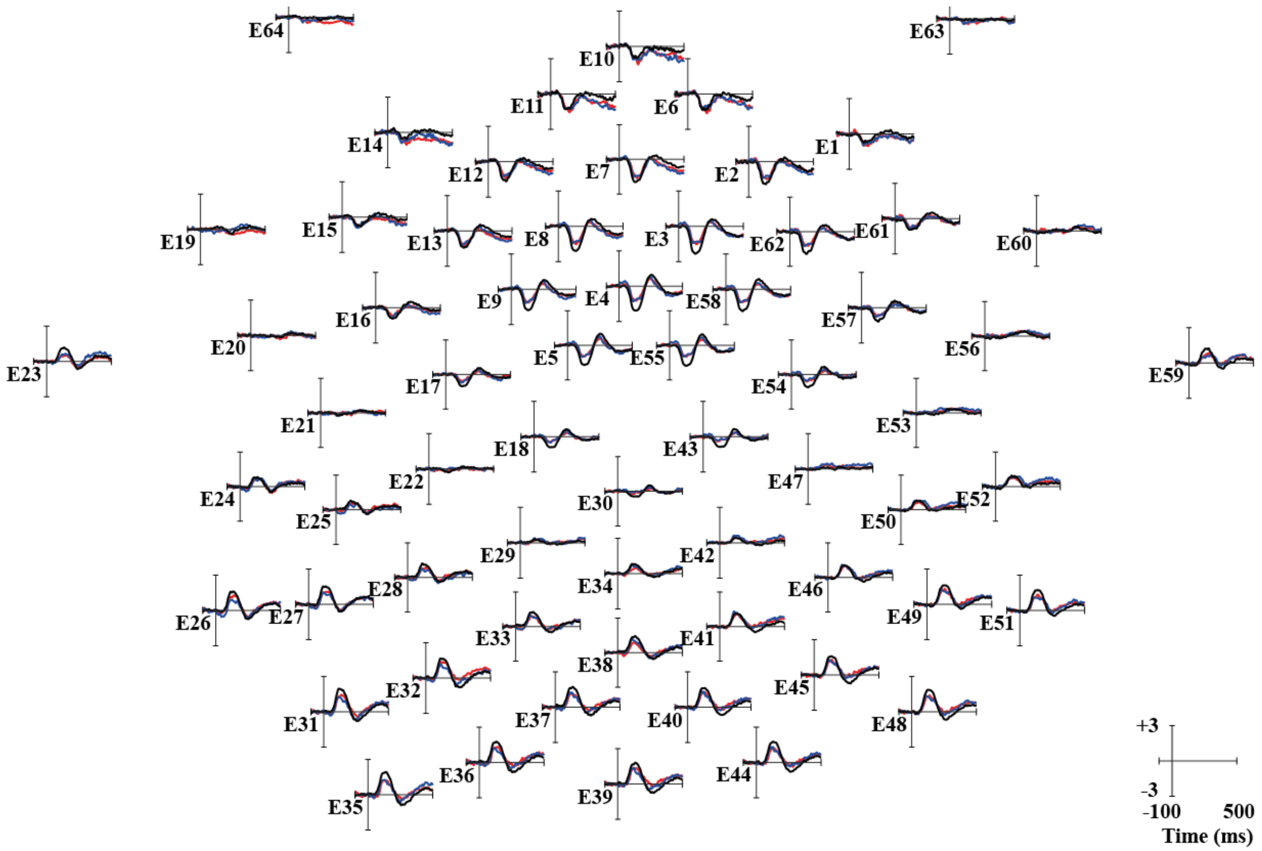

Figure S4: Frequency mismatch negativity (fMMN) at all 64 channels of the three groups. Black, blue, and red curves indicate healthy controls (HC), ultra-high risk (UHR) individuals, and patients with psychotic disorders (PD), respectively.

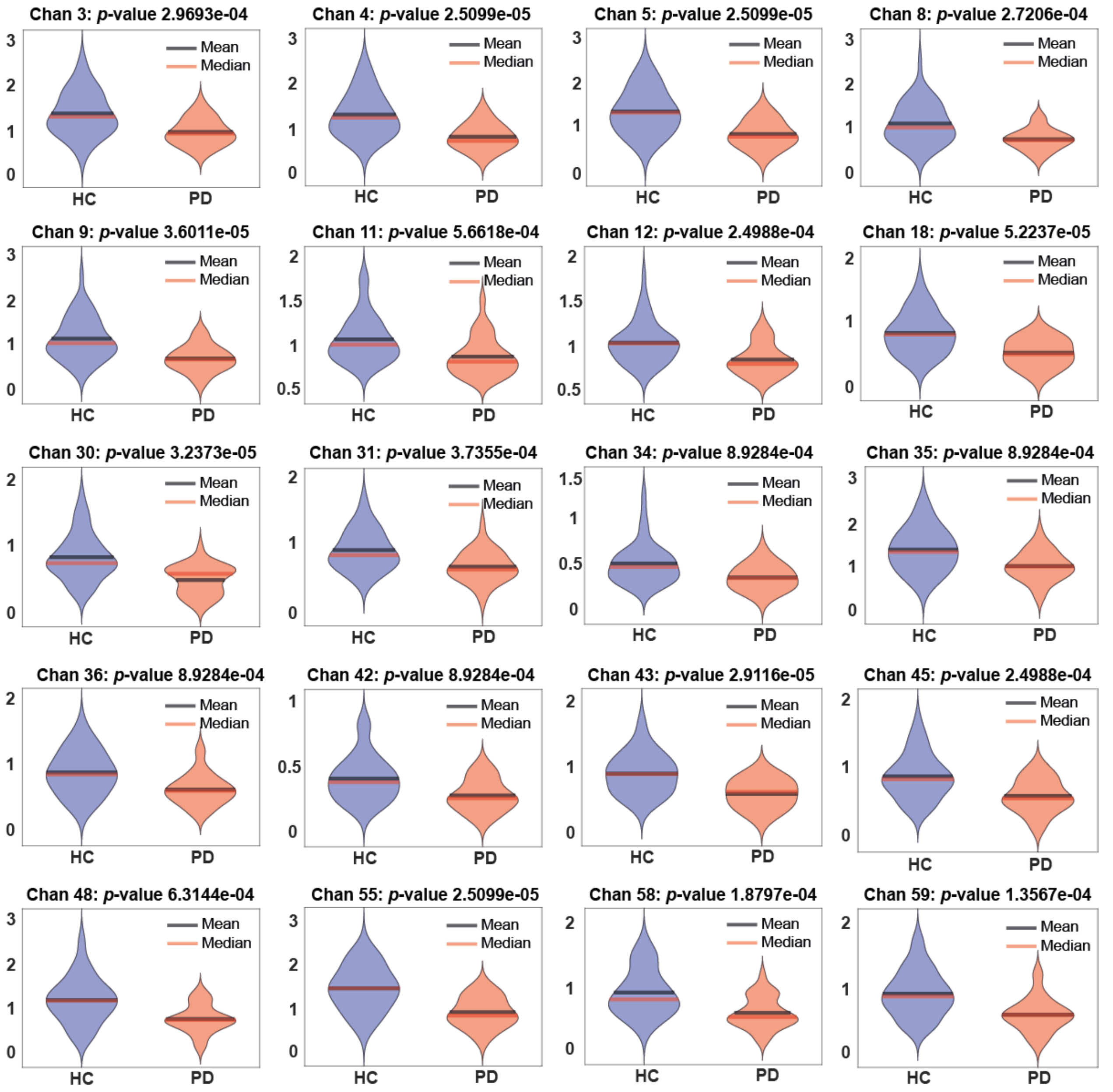

Figure S5: Violin plots of 20 electrodes with significantly increasing standard deviations in the HC group compared with those in the PD group. The experiment was conducted under duration deviant (dD) stimuli. The p-values were calculated using the one-sided paired-sample t-test with FDR correction. Nodes were selected at $p < 10^{-3}$.

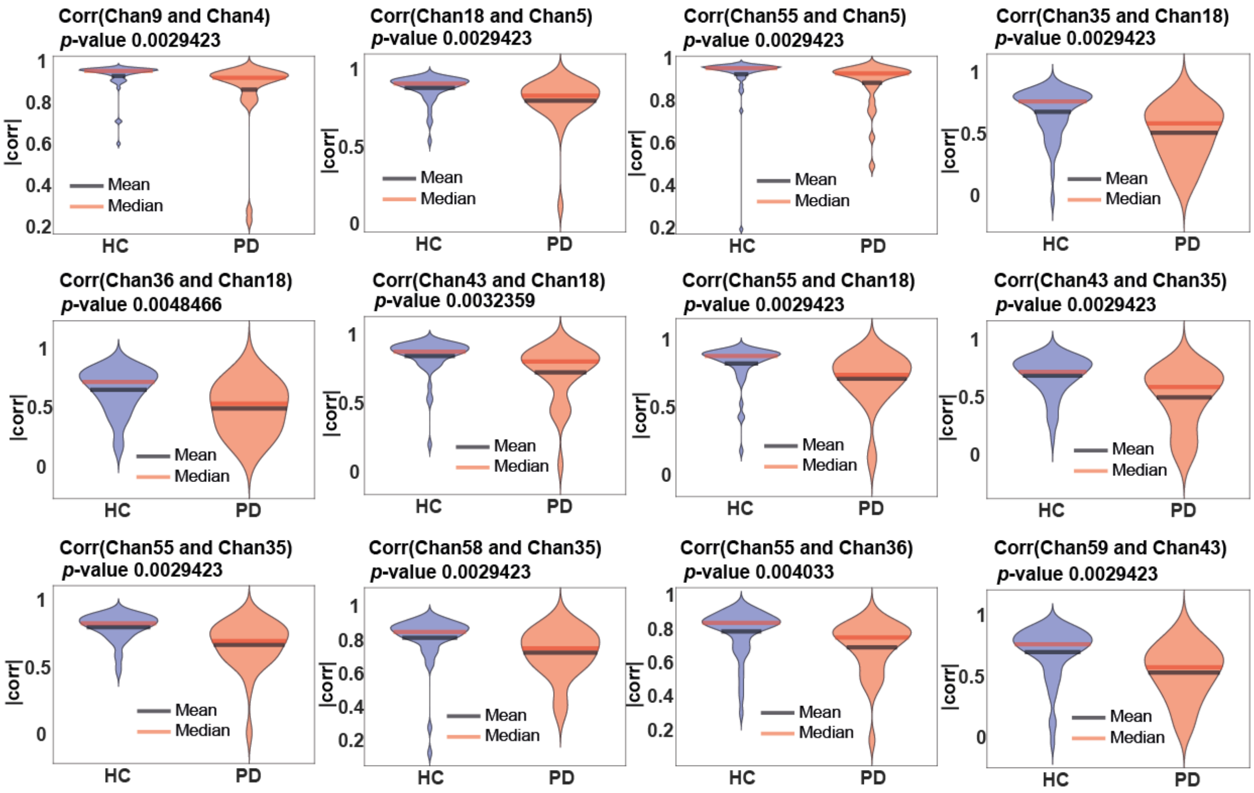

Figure S6: Violin plots of 12 edges with significantly increasing correlations in the HC group compared with those in the PD group. The experiment was conducted under duration deviant (dD) stimuli. The p-values were calculated using the one-sided Wilcoxon rank-sum test with FDR correction. Edges were selected at $p < 0.005$.

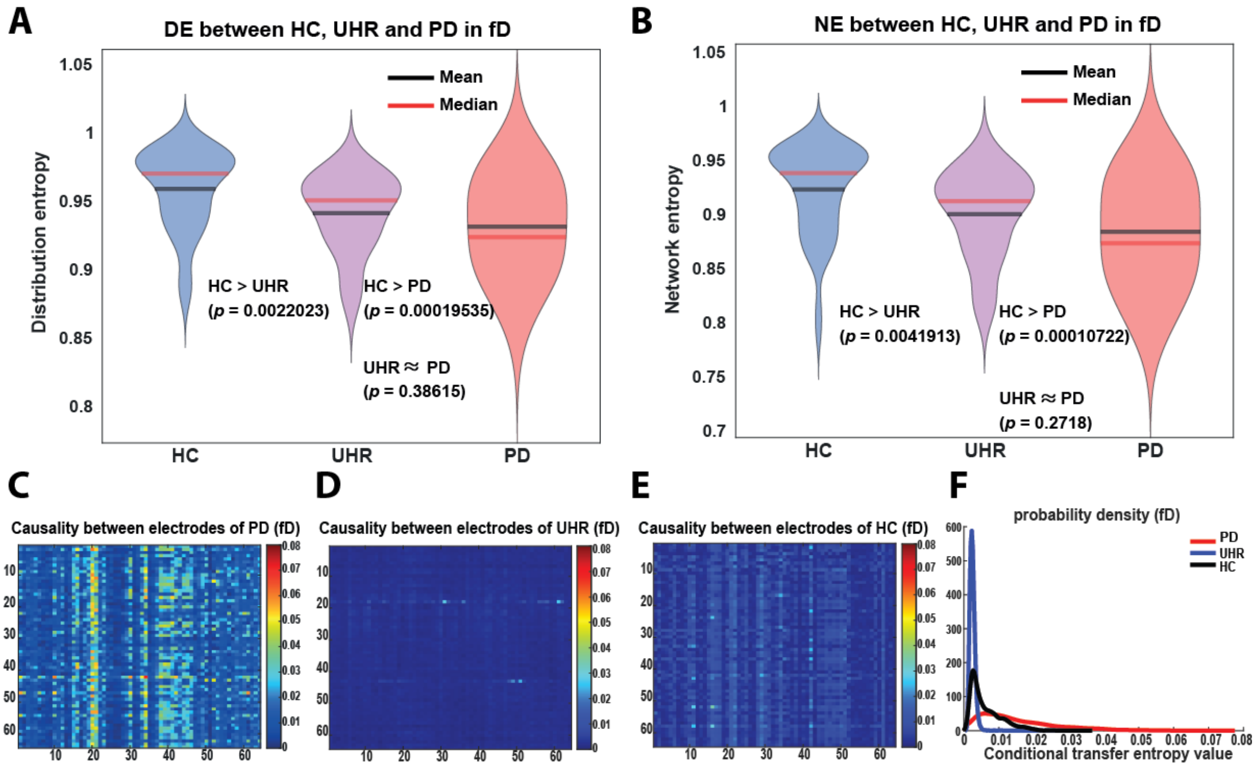

Figure S7: Entropy and causality difference between the HC, UHR, and PD groups in the frequency deviant (fD) case. (A) Violin plot for the distribution entropy of mutual information between each pair of nodes. (B) Violin plot for the network entropy of mutual information between each pair of nodes. For "HC > UHR" and "HC > PD", p-values were calculated by the one-sided Wilcoxon rank-sum test, whereas for "UHR ≈ PD", the two-sided Wilcoxon rank-sum test was used. The HC group exhibited significantly higher entropy than the PD and UHR groups, which did not significantly differ. (C-E) Heatmaps of the direct causality between 64 electrodes measured by the conditional transfer entropy (CTE). (F) Probability density functions of the CTE. On average, the PD has much stronger direct causalities between electrodes than the HC/UHR.

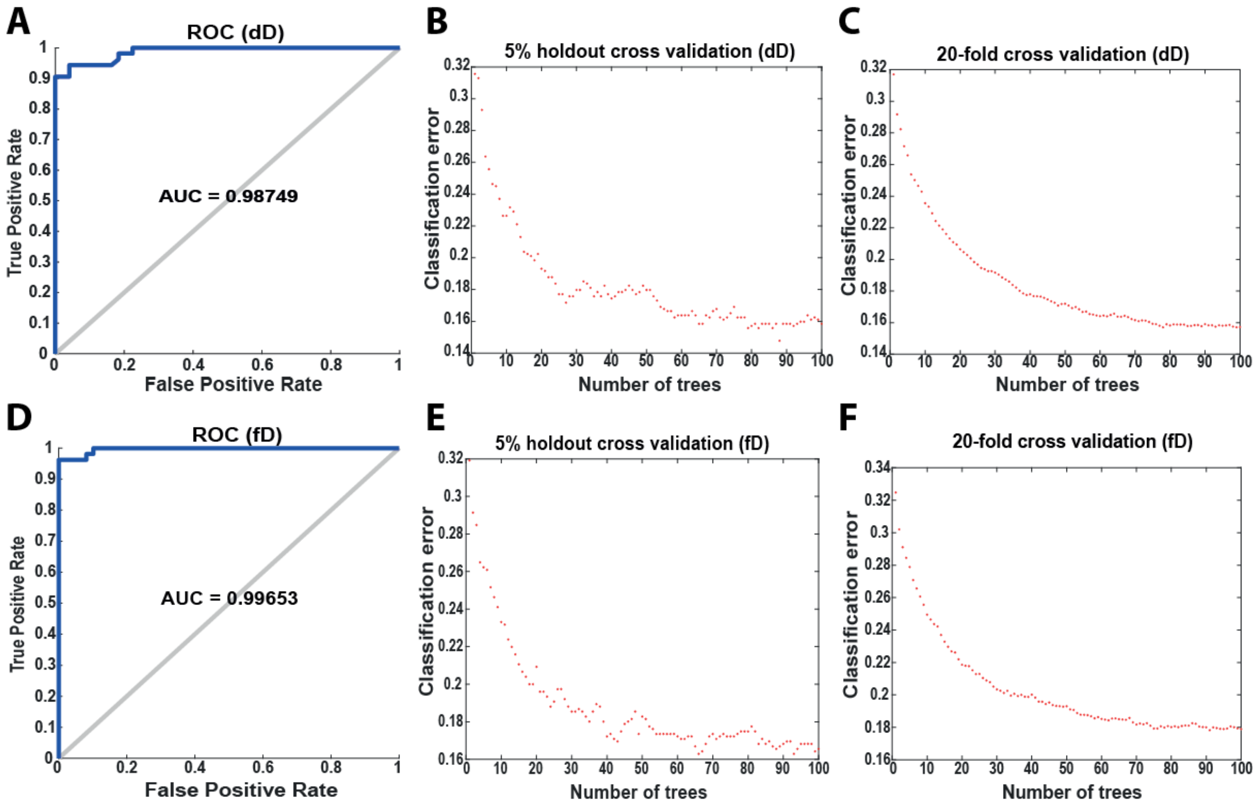

Figure S8: Ensemble classification characteristics for the duration deviant experiment (dD) dataset and frequency deviant experiment (fD) dataset with DNM features. (A) and (D) Receiver operating characteristic (ROC) curves for the epochs data in dD and fD, respectively. (B) and (E) The 5% holdout loss cross validations. (C) and (F) The 20-fold loss cross validations for the corresponding classifiers. In (B), the loss is approximately 0.1585 (accuracy 0.8415), and in (C), the loss tends to be 0.1572 (accuracy 0.8428). In (E), the loss is approximately 0.1656 (accuracy 0.8344), and in (F), loss tends to be 0.1789 (accuracy 0.8211).

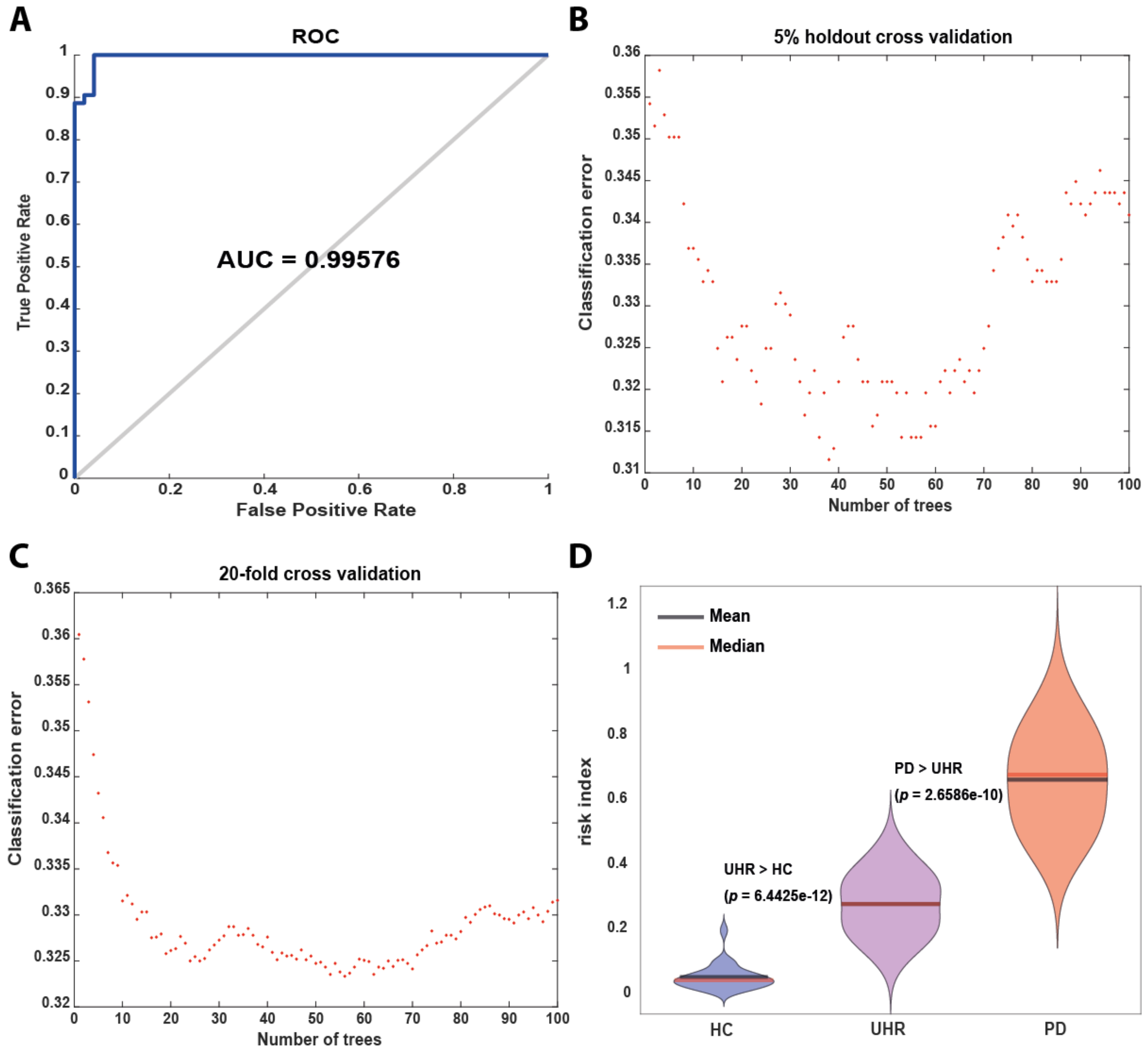

Figure S9: Properties of the ensemble classifier with dMMN amplitude values of each epoch as features. (A) Receiver operating characteristic (ROC) curve for epochs data in dD. (B) The 5% holdout loss and (C) 20-fold loss for cross validation of the classifier. In (B), the loss is approximately 0.2222 (accuracy 0.7778), and in (C), the loss tends to be 0.2108 (accuracy 0.7892). (D) Violin plot of risks for the three groups. HC has the lowest risk and FES has the highest risk, whereas UHR exhibits medial risk. The one-sided Wilcoxon rank-sum test was used for determining the significance of difference between groups, and the p-values are listed in (D).

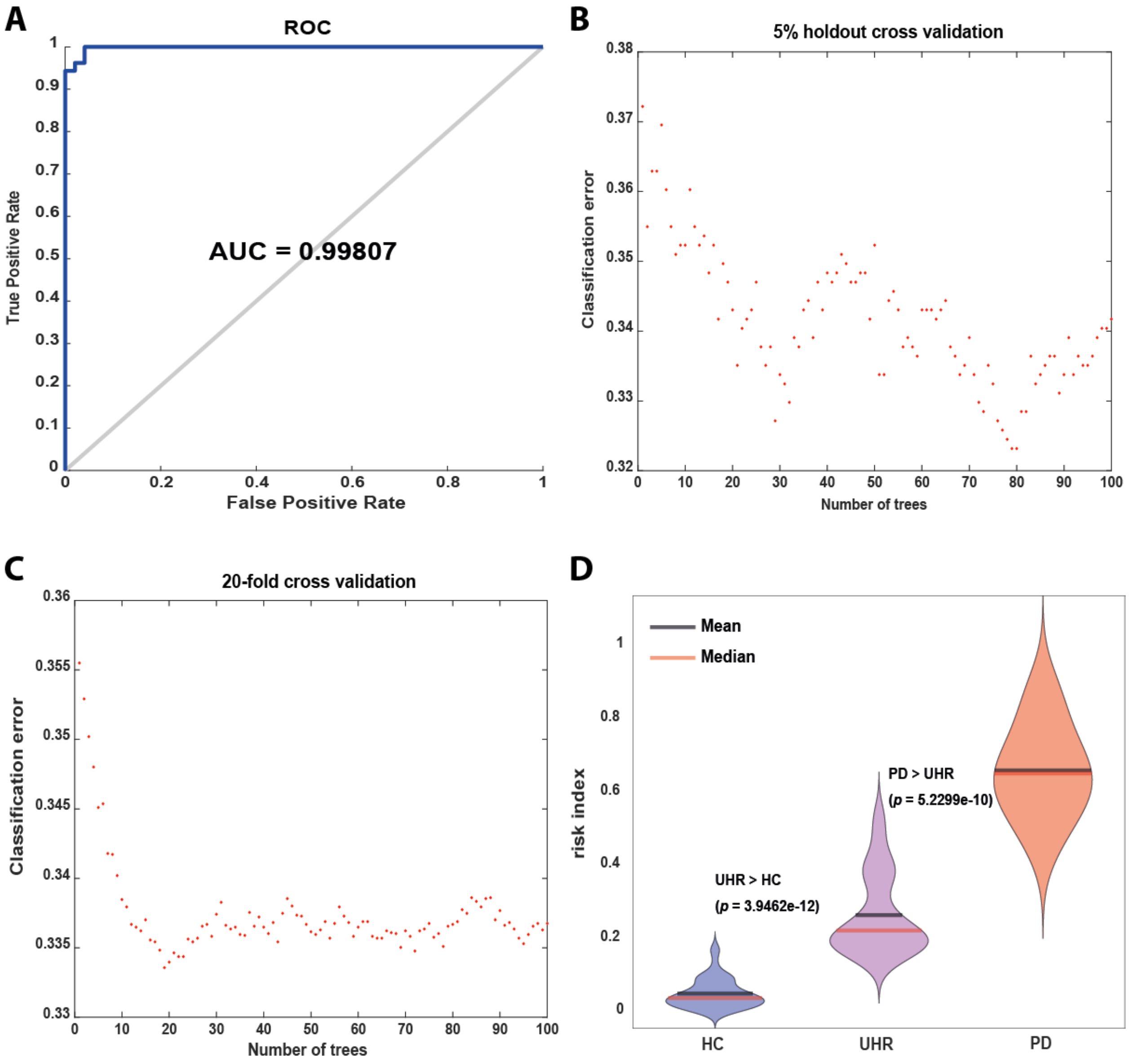

Figure S10: Properties of the ensemble classifier with fMMN amplitude values of each epoch as features. (A) Receiver operating characteristic (ROC) curve for epochs data in fD. (B) The 5% holdout loss and (C) 20-fold loss for cross validation of the classifier. In (B), the loss is approximately 0.1686 (accuracy 0.8314), and in (C), the loss tends to be 0.2025 (accuracy 0.7975). (D) Violin plot of risks for the three groups. HC has the lowest risk and FES has the highest risk, whereas UHR exhibits medial risk. The one-sided Wilcoxon rank-sum test was used for determining the significance of difference between groups, and the p-values are listed in (D).

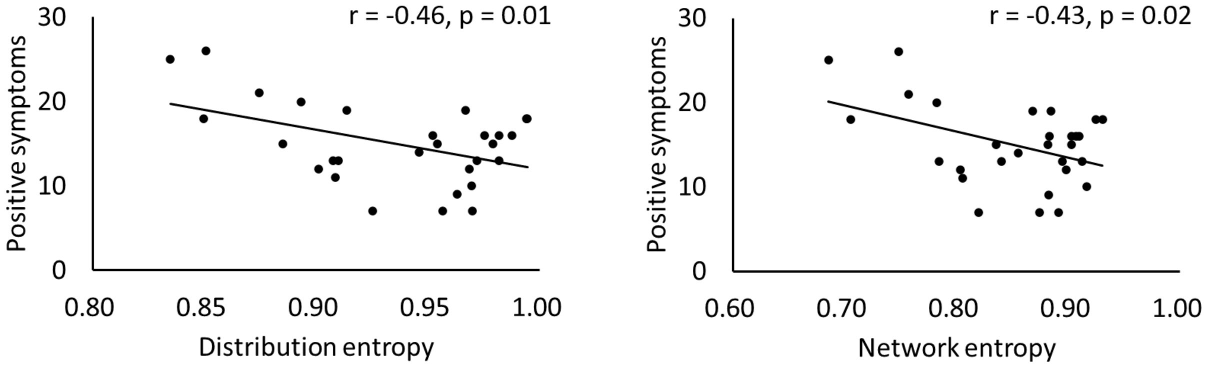

Figure S11: Correlations of positive symptoms with the distribution entropy (left) and network entropy (right). The 29 samples are from the psychotic disorder (PD) group, and entropies are calculated under duration deviant experiments. The worse the positive symptoms are, the stable is the network, as low entropy implies stability.

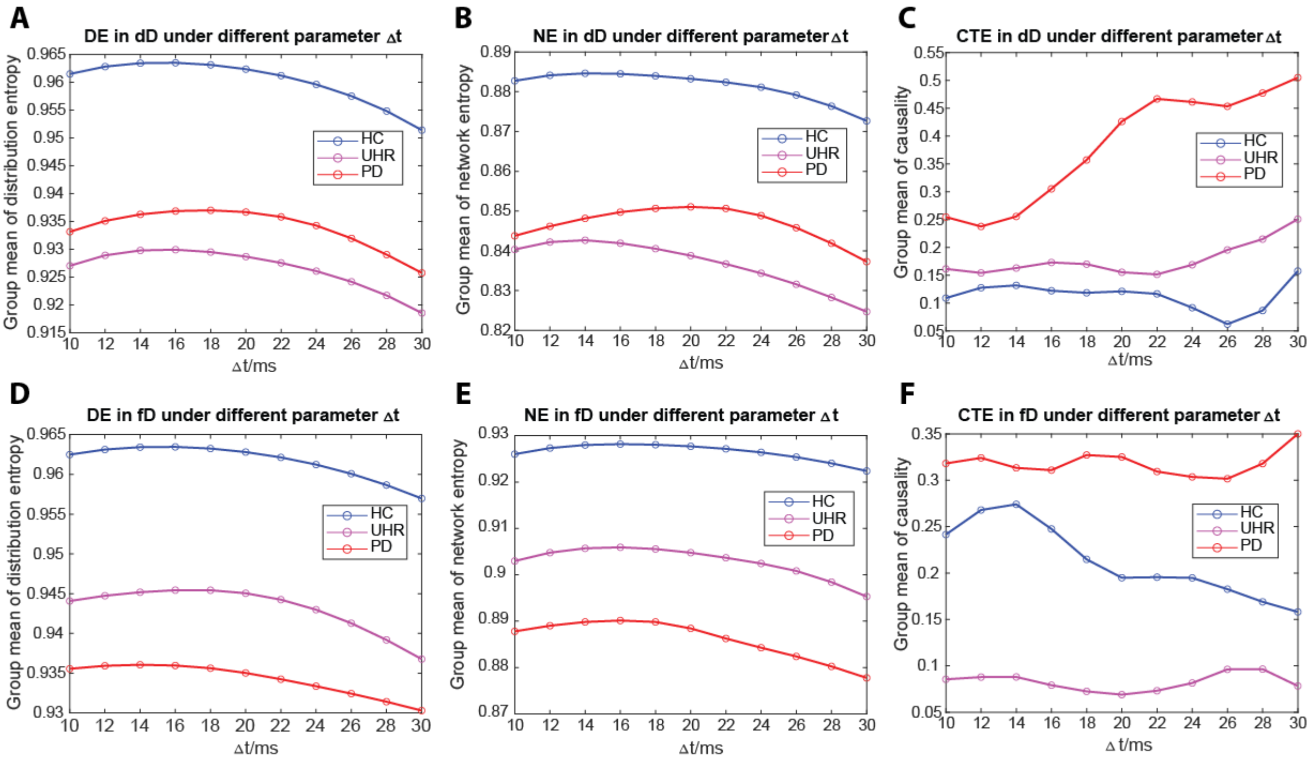

Figure S12: Group means of the DE, the NE, and the averaged CTE are calculated under different time intervals Δt. (A-C) are in the dD case, and (D-F) are in the fD case. Δt varies from 10ms to 30ms. The orders of three groups (the HC, the UHR, and the PD) are not sensitively influenced by Δt.

---

[1] Koike S, Takano Y, Iwashiro N, Satomura Y, Suga M, Nagai T, *et al.* A multimodal approach to investigate biomarkers for psychosis in a clinical setting: the integrative neuroimaging studies in schizophrenia targeting for early intervention and prevention (IN-STEP) project. *Schizophr Res* 2013; 143(1): 116-124.

[2] Nagai T, Tada M, Kirihara K, Yahata N, Hashimoto R, Araki T, *et al.* Auditory mismatch negativity and P3a in response to duration and frequency changes in the early stages of psychosis. *Schizophr Res* 2013; 150(2-3): 547-554.
[3] Koshiyama D, Kirihara K, Tada M, Nagai T, Koike S, Suga M, *et al.* Duration and frequency mismatch negativity shows no progressive reduction in early stages of psychosis. *Schizophr Res* 2017; 190: 32-38.
[4] Nagai T, Kirihara K, Tada M, Koshiyama D, Koike S, Suga M, *et al.* Reduced mismatch negativity is associated with increased plasma level of glutamate in first-episode psychosis. *Sci Rep* 2017; 7(1): 1-9.
[5] Koshiyama D, Fukunaga M, Okada N, Yamashita F, Yamamori H, Yasuda Y, *et al.* Role of subcortical structures on cognitive and social function in schizophrenia. *Sci Rep* 2018; 8(1): 1-9.
[6] Koshiyama D, Fukunaga M, Okada N, Yamashita F, Yamamori H, Yasuda Y, *et al.* Subcortical association with memory performance in schizophrenia: a structural magnetic resonance imaging study. *Transl Psychiatry* 2018; 8(1): 1-11.

[7] Kobayashi H, Nozaki S, Mizuno M. Reliability of the Structured Interview for Prodromal Syndromes Japanese version (SIPS-J). *Jpn Bull Soc Psychiatry* 2007; 1: 168-174.

[8] Diagnostic and Statistical Manual of Mental Disorders. Fourth Edition (DSM-IV). American Psychiatric Association; 1994.

[9] Matsuoka K, Kim Y. Japanese Adult Reading Test (JART). Shinkou-Igaku Publishers, Tokyo (In Japanese); 2006.

[10] Matsuoka K, Uno M, Kasai K, Koyama K, Kim Y. Estimation of premorbid IQ in individuals with Alzheimer's disease using Japanese ideographic script (Kanji) compound words: Japanese version of National Adult Reading Test. *Psychiatry Clin Neurosci* 2006; 60(3): 332–339.

[11] Kay SR, Fiszbein A, Opler LA. The positive and negative syndrome scale (PANSS) for schizophrenia. *Schizophr Bull* 1987; 13(2): 261–276.

[12] Inada T, Inagaki A. Psychotropic dose equivalence in Japan. *Psychiatry Clin Neurosci* 2015; 69(8): 440-447.

[13] Delorme A, Makeig S. EEGLAB: an open source toolbox for analysis of single-trial EEG dynamics including independent component analysis. *J Neurosci Methods* 2004; 134(1): 9-21.

[14] Chang CY, Hsu SH, Pion-Tonachini L, Jung TP. Evaluation of artifact subspace reconstruction for automatic EEG artifact removal. In: *Proceedings of the 40th Annual International Conference of the IEEE Engineering in Medicine and Biology Society (EMBC)*. IEEE; 2018, p. 1242-1245.
